# Bringing severe acute malnutrition treatment close to households through community health workers can lead to early admissions and improved discharge outcomes

**DOI:** 10.1371/journal.pone.0227939

**Published:** 2020-02-05

**Authors:** Noemí López-Ejeda, Pilar Charle-Cuellar, Franck G. B. Alé, José Luis Álvarez, Antonio Vargas, Saul Guerrero

**Affiliations:** 1 Action Against Hunger, Madrid, Spain; 2 EPINUT Research Group (ref. 920325), Complutense University of Madrid, Madrid, Spain; 3 Action Against Hunger, Bamako, Mali; 4 Action Against Hunger, London, England; 5 Action Against Hunger, New York, New York, United States of America; University of Ghana, GHANA

## Abstract

Severe acute malnutrition (SAM) affects over 16.6 million children worldwide. The integrated Community Case Management (iCCM) strategy seeks to improve essential health by means of nonmedical community health workers (CHWs) who treat the deadliest infectious diseases in remote rural areas where there is no nearby health center. The objective of this study was to assess whether SAM treatment delivered by CHWs close to families’ locations may improve the early identification of cases compared to outpatient treatment at health facilities (HFs), with a decreased number complicated cases referred to stabilization centers, increased anthropometric measurements at admission (closer to the admission threshold) and similarity in clinical outcomes (cure, death, and default). The study included 930 children aged 6 to 59 months suffering from SAM in the Kita district of the Kayes Region in Mali; 552 children were treated by trained CHWs. Anthropometric measurements, the presence of edema, and other medical signs were recorded at admission, and the length of stay and clinical outcomes were recorded at discharge. The results showed fewer children with edema at admission in the CHW group than in the HF group (0.4% vs. 3.7%; OR = 10.585 [2.222–50.416], p = 0.003). Anthropometric measurements at admission were higher in the CHW group, with fewer children falling into the lowest quartiles of both weight-for-height z-scores (20.2% vs. 31.5%; p = 0.002) and mid-upper arm circumference (18.0% vs. 32.4%; p<0.001), than in the HF group. There was no difference in the length of stay. More children in the CHW group were cured (95.9% vs. 88.7%; RR = 3.311 [1.772–6.185]; p<0.001), and there were fewer defaulters (3.7% vs. 9.8%; RR = 3.345 [1.702–6.577]; p<0.001) than in the HF group. Regression analyses demonstrated that less severe anthropometric measurements at admission resulted in an increased probability of cure at discharge. The study results also showed that CHWs provided more integrated care, as they diagnosed and treated significantly more cases of infectious diseases than HFs (diarrhea: 36.0% vs. 18.3%, p<0.001; malaria: 41.7% vs. 19.8%, p<0.001; acute respiratory infection: 34.8% vs. 25.2%, p = 0.007). The addition of SAM treatment in the curative tasks that the CHWs provided to the families resulted in earlier admission and more integrated care for children than those associated with HFs. CHW treatment also achieved better discharge outcomes than standard community treatment.

## Introduction

Severe acute malnutrition (SAM) is the most extreme and visible form of undernutrition, and children suffering from it require urgent treatment [1]. Severely acutely malnourished children are 11.63 times more likely to die than those who are well-nourished [2]. It is estimated that there are currently 16.6 million children under the age of 5 with severe wasting (2.4% worldwide) [3]. Over the last four decades, SAM treatment has shifted from small-scale inpatient treatment, which reached just 4–10% of affected children [4], to outpatient therapeutic feeding programs under the Community-based Management of Acute Malnutrition (CMAM) protocol [5] at health facilities (HFs), which has increased coverage to almost 40% [6].

This outpatient approach relies on the use of ready-to-use therapeutic food (RUTF), with a nutritional composition similar to therapeutic milk formulas used in the inpatient treatment, but it does not require either refrigeration or water to be prepared and can be safely consumed at the household level. The results of a systematic review and meta-analysis showed that children treated through this community approach were 51% more likely to recover than those treated at hospitals [7].

The Integrated Community Case Management (iCCM) strategy was developed to improve access to essential health services for children in hard-to-reach rural areas. Under the general iCCM protocol, pneumonia, diarrhea, and malaria are treated by nonmedical community health workers (CHWs) in their own villages. These CHWs are also encouraged to identify SAM cases through the assessment of mid-upper arm circumference (MUAC), but in most cases, they are not allowed to treat SAM [8]. All severely acutely malnourished children without other severe medical complications identified by the CHWs are referred to the nearest HFs to be admitted to a CMAM program. However, the effectiveness of these referrals is unknown, as they are seldom reported. A review on the coverage of CMAM programs in 21 low- and middle-income countries has identified the distance to the HFs as a main barrier for families to access SAM treatment [6].

Since 2010, efforts have been made to add SAM treatment to the curative tasks that CHWs can provide close to families, referred to as the iCCM+ approach. A review of the operational experiences of this approach has recently been published [9] and shows that programs implementing this new community approach can achieve better outcomes in terms of recovery and defaulter rates than standard CMAM programs performed at HFs far away from the affected villages. This review also found that the iCCM+ model could potentially double the coverage of SAM treatment services, achieving over 80% treatment coverage in a cost-effective manner, reducing the time and money that the families would expend to treat their children at the health centers, which are usually located far away from their households [10,11].

The first general review of SAM management published by Collins et al. in 2006 [4] stated that the severity and prognosis of SAM and the determinants of successful treatment are primarily dependent on the time of presentation. The increase in coverage achieved by the CHW-delivered approach was hypothesized to bring about improvements in early presentation or admissions when children have relatively less severe forms of the condition and few medical complications [9].

In 2018, Mali had 4.1 million people in need of humanitarian assistance, and almost one in five Malians was in a state of food insecurity, which is an increase from 2.5 million in 2016 [12]. According to the Human Development Index, Mali is the 182th poorest country in the world out of a total of 189 countries [13], and global acute malnutrition affects 10.7% of children, 2.6% of whom are severely acutely malnourished. Outside the conflict zone, the Kayes Region has the highest prevalence of global acute malnutrition (14.2% with a 2.6% SAM) due to erratic rains that limit dietary diversity, poor hygiene and sanitation conditions, and inadequate access to clean water [13, 14]. In 2010, the Malian government approved the iCCM of essential care to be delivered by CHWs (*Agents de Santé Communautaire*) in villages located 5 km or more from a health center. Each CHW has a catchment area of approximately 700 people from 2–3 villages, and they are supported by a volunteer cadre (*Relais Communautaire*) who are in charge of screening and referrals, as well as communication activities aimed at effecting social and behavioral change. In 2016, there were 2,377 active CHWs, but it is estimated that 4,876 are needed for full health coverage [15].

The study aimed to assess whether providing treatment close to families through CHWs (by including SAM management in the curative services of the iCCM protocol they provide) could diminish severity at admission, leading to better discharge outcomes than those associated with the CMAM standard outpatient treatment already provided in the health centers typically located far away from the families in a rural area of the Kayes Region in Mali.

## Methods

The sample consisted of 930 children aged 6 to 59 months suffering from SAM in seven communes of the Kita district of the Kayes Region in southwest Mali. All the children were enrolled in the multicenter and prospective study, with the aim of exploring the potential of including SAM treatment as part of the services delivered by CHWs to improve affected family access and coverage. The study was approved by the Ethical Committee of the National Institute for Research in Public Health (*Institute National de la Recherche en Santé Publique*, *INRSP*). The intervention was carried out for twelve months between February 2015 and February 2016.

The original study was carried out in two distinct health areas. In one of them, the standard CMAM protocol was applied whereby that severely acutely malnourished children were treated only in the HFs (control group: 4 HFs). In the other area, the iCCM+ protocol was applied; in addition to HFs, treatment by CHWs close to the households was added (intervention group: 3 HFs + 17 CHWs). The results of the coverage and effectiveness of this combined outpatient treatment model with medical staff at HFs plus CHWs at the household level have been previously published elsewhere [16]. According to Malian protocols, CHWs previously received two weeks of training from health staff on iCCM and CMAM techniques, with one month of on-site practice at the HF. During the study period, they received a 3-day refresher training and attended monthly meetings at the HF. Their sociodemographic characteristics and the quality of care provided are published elsewhere [17].

The present work is based on a secondary data analysis of previous study data consisting of the disaggregation of children by their treatment provider independent of the health area (HF vs. CHW), with the aim to assess whether providing treatment close to households through CHWs allows children to be admitted into treatment earlier and in a relatively less severe condition, with a positive impact on the treatment outcomes compared to standard care at the HFs. Accordingly, the intervention group consisted of all the children assessed and treated by the 17 CHWs in their communes (Tambaga, Bougarabaya, and Kobiri) and the comparison control group consisted of all the children assessed and treated in the seven HFs that covered the same three communes, along with four others (Guenikoro, Kassaro, Dafela, and Sebekoro). Both treatment providers used the same tools and followed the same protocols and criteria for initial assessment, admission, follow-up, and discharge of the children according to the National requirements; thus, the main difference between the two treatment models was the convenience of accessing CHWs (CHWs typically assess and treat children closer to the households compared to HFs) and the education degree of the providers (nonmedical CHWs compared to doctors or nurses at HFs).

At the time of admission, all the children were assessed for edema, diarrhea, vomiting, fever, cough, malaria, dermatosis, and conjunctival pallor. A malaria test was also performed in children with a temperature above 38°C or suspected of having the disease. Patients with severe medical complications or children who were not able to successfully complete an appetite test were referred from the CHW site or the HF to stabilization centers for inpatient treatment. Anthropometric assessments were also performed to evaluate weight (kg), height (cm), and the MUAC (mm). The inclusion criteria in both groups were those recommended by the World Health Organization (WHO) [18]: the presence of mild bilateral edema or a MUAC under 115 mm or weight-for-height z-score (WHZ) less than -3 according to the WHO growth reference [19].

All children meeting those criteria received weekly rations of RUTF according to their weight (170 Kcal/kg/day). Recovery was defined as the absence of edema and two consecutive weekly WHZ measurements above -1.5 or MUAC readings above 125 mm. Other reasons for exclusion included death, nonresponse (failure to gain weight within 14 days or weight loss during the first seven days after admission or during two consecutive follow-up visits or a total loss of 5% at any time during treatment) and default (absence at two consecutive weekly visits). The length of stay was calculated as the days elapsed between the admission and discharge dates. To ensure treatment quality, both the CHWs and medical personnel in the HFs were supervised twice a month by specialized Action Against Hunger staff and every three months by the project’s technical committee together with the staff of the INRSP.

Statistical analyses were performed with SPSS v.25 software. The normal distribution of quantitative variables was assessed with the Kolmogorov-Smirnoff test with Lilliefors correction. All variables showed a nonnormal distribution, so the Mann-Whitney test was applied to compare their central tendency and dispersion parameters. For proportion comparisons, a chi-square test was used, and Yates’s correction or the Monte Carlo exact test was applied when the minimum expected count for a category was fewer than 5 cases. For variables related to the admission moment, a logistic regression analysis was carried out to assess crude and adjusted odds ratios (ORs), and for those related to stay and discharge, the ORs were estimated applying the Cochran-Mantel-Haenszel method to adjust for confounders.

## Results

A total of 552 children (59.4% of the total) were treated by CHWs in their communities, and 378 children (40.6%) received traditional outpatient treatment by medical staff in the health facilities. The comparison groups did not differ in terms of sex ratios (CHWs: 58.2% female; HFs = 56.6% female; p = 0.641) or age (mean age: CHWs 14.58 ± 8.46 months vs. HFs 14.15 ± 7.67 months, p = 0.698; ≤12 months: CHWs 56.2% vs. 59.5%, p = 0.308). Table 1 shows the distribution of the children according to admission type. More children were new admissions in the HF group than in the CHW group, while readmissions (children re-enrolled after abandonment with an absence of less than two months), relapses (children re-enrolled after more than two months of absence or after being discharged as cured), and transfers from stabilization centers were more prevalent in the CHW group than in the HF group.

**Table 1:** Distribution of children according to the reason for admission by each model of outpatient treatment.

	Community Health Workers		Health Facilities		Comparison (p value)
	n	%	n	%	
New admission	462	83.7	347	91.8	<0.001
Readmission	13	2.4	2	0.5	0.030
Relapse	25	4.5	7	1.9	0.028
Transfer from URENAS	9	1.6	5	1.3	0.705^NS^
Transfer from URENI	43	7.8	17	4.5	0.045

^NS^: No significant difference / URENAS: *Unité de Récupération et d’Education Nutritionnelle* = Unit for outpatient severe nutritional recovery and education; URENI: Unité de Récupération et d’Education Nutritionnelle Intensive = Unit for intensive nutritional recovery and education.

The results of the medical assessments at admission are shown in Table 2. There was no difference between the models in the proportion of children who had to be referred to stabilization centers due to severe medical complications. However, 3.7% of the children in the HF group were affected by edema, which was significantly higher than 0.4% in the CHW group. After adjusting for sex and age, the probability of edema at admission was markedly higher in children admitted for treatment at a health center than those presenting for treatment by a CHW (OR = 10.585, 95% confidence interval (C.I.) [2.222–50.416], p = 0.003). In contrast, diarrhea (OR = 1.512, 95% C.I. [1.049–2.179], p = 0.027) and malaria (OR = 2.554. 95% C.I. [1.838–3.548], p<0.001) were more prevalent in children assessed by CHWs than in children admitted to HFs. In both groups, approximately one-quarter of the sample reported coughing, and one-third had a fever.

**Table 2:** Presence of disease signs during the medical assessment at admission according to the two models of outpatient treatment.

	Community Health Worker treatment		Health Facility treatment		Comparison (p value)
	n	%	n	%	
Referred at admission*	28	5.1	22	5.9	0.615
Edema	2	0.4	14	3.7	<0.001
Other signs:					
Diarrhea	104	18.8	51	13.5	0.032
Vomiting	52	9.4	47	12.4	0.143^NS^
Fever	195	35.3	123	32.5	0.379^NS^
Cough	153	27.7	93	24.6	0.290^NS^
Dermatosis	0	0.0	4	1.1	0.056^NS^
Pale Conjunctiva	2	0.4	12	3.2	0.001
Malaria tests performed:	511	92.6	319	84.4	
Positive results	201	39.3	65	20.4	<0.001

*Children who were referred to the URENI (*Unité de Récupération et d’Education Nutritionnelle Intensive* = Unit for intensive nutritional recovery and education) / NS: Not significant difference

**Fig 1:**
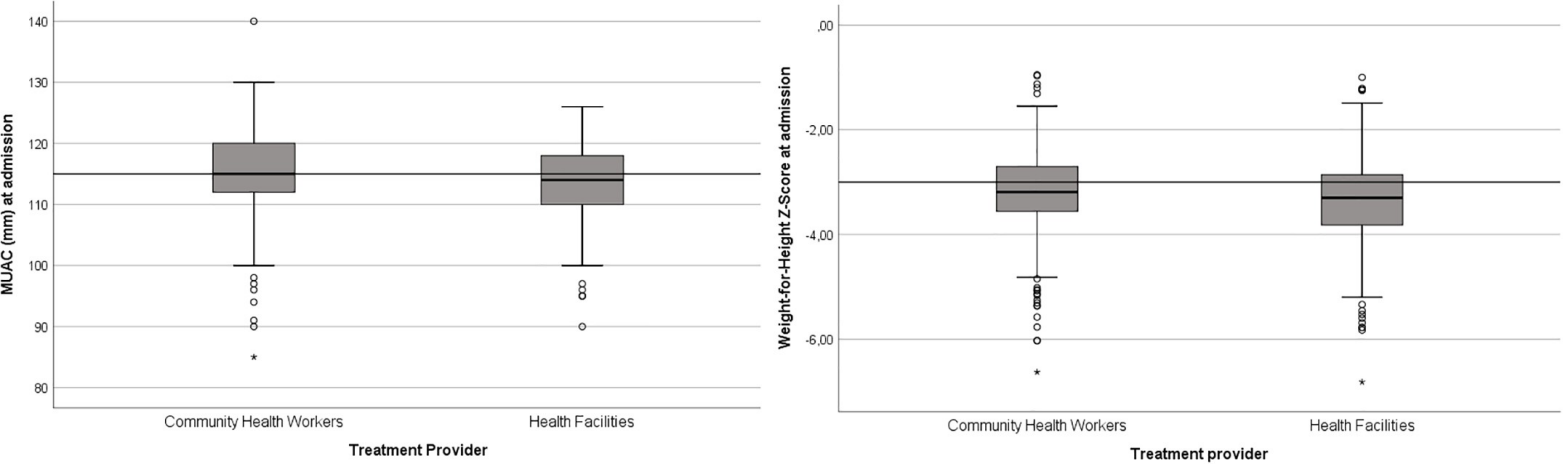
Anthropometric measurements of the children at admission according to the two models of outpatient treatment. Inclusion criteria for severe acute malnutrition treatment is marked with a discontinuous line.

The analysis of anthropometric measurements at admission included only new cases and relapse cases, given that children requiring readmission or transfer had already started their treatment, which could influence their measurements at readmission time. Children with edema were also been excluded from this analysis since their WHZ was affected. Fig 1 shows the comparison of WHZ and MUAC at admission according to the two models. The HF group had a slightly lower weight (6.30 ± 1.04 kg vs. 6.49 ± 1.14 kg; p = 0.026) and slightly lower WHZ (-3.40 ± 0.82 vs. -3.23 ± 0.75; p<0.001) than the CHW group, although the groups did not differ in height (HFs 70.59 ± 6.10 cm; CHWs 71.13 ± 6.44 cm; p = 0.340).

**Fig 2:**
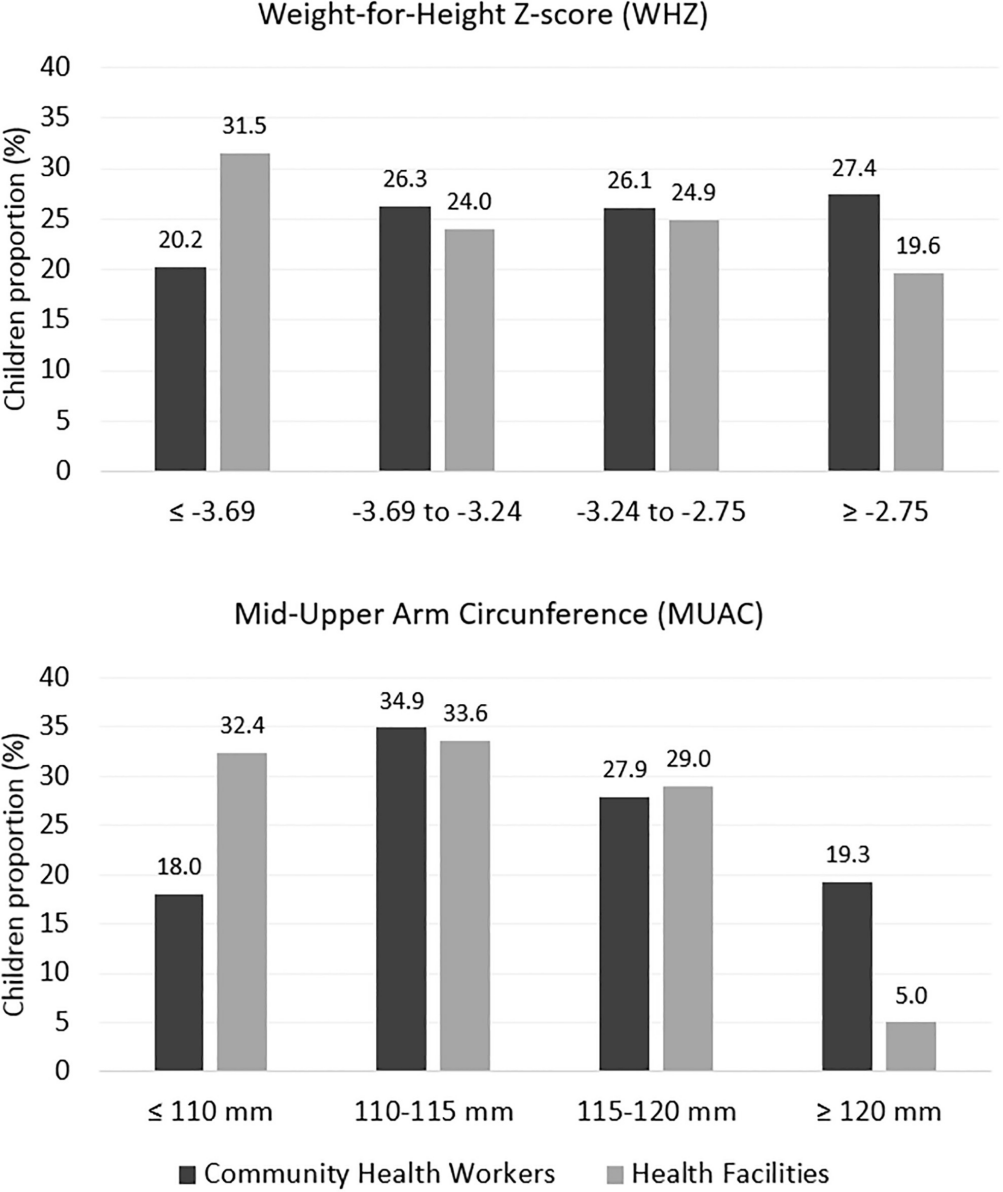
Distribution of children according to the quartiles of MUAC and WHZ at admission.

Fig 2 shows the distribution of the children according to WHZ quartiles calculated for the whole sample. The proportion of children who fell into the lowest quartile was higher in the HF group (p = 0.002) than in the CHW group. Considering only those children admitted with a WHZ < -3 (n = 498), the proportion with the most severe condition (≤ -3.69 z-score) was also higher in the HF group than in the CHW group (44.7% vs. 33.8%, p = 0.013; OR adjusted by sex and age = 1.596, 95% C.I. [1.107–2.302], p = 0.012).

Similar results were found for MUAC at admission. The median values differed between the models, with the HF group showing the lowest value, at 114 mm [interquartile range (IQR) 110–118], compared to 115 mm [IQR 112–120] in the CHW model (p<0.001). Fig 2 also shows that the proportion of children who fell into the lowest quartile for MUAC in the HF group was almost double that in the CHW group (p<0.001). Considering only those children with a MUAC <115 mm (n = 378), the proportion with the most severe condition (≤110 mm) was markedly higher in the HF group than in the CHW group (60.8% vs. 39.6%, p<0.001; OR adjusted by sex and age = 2.350, 95% C.I. [1.551–3.563], p<0.001).

**Fig 3:**
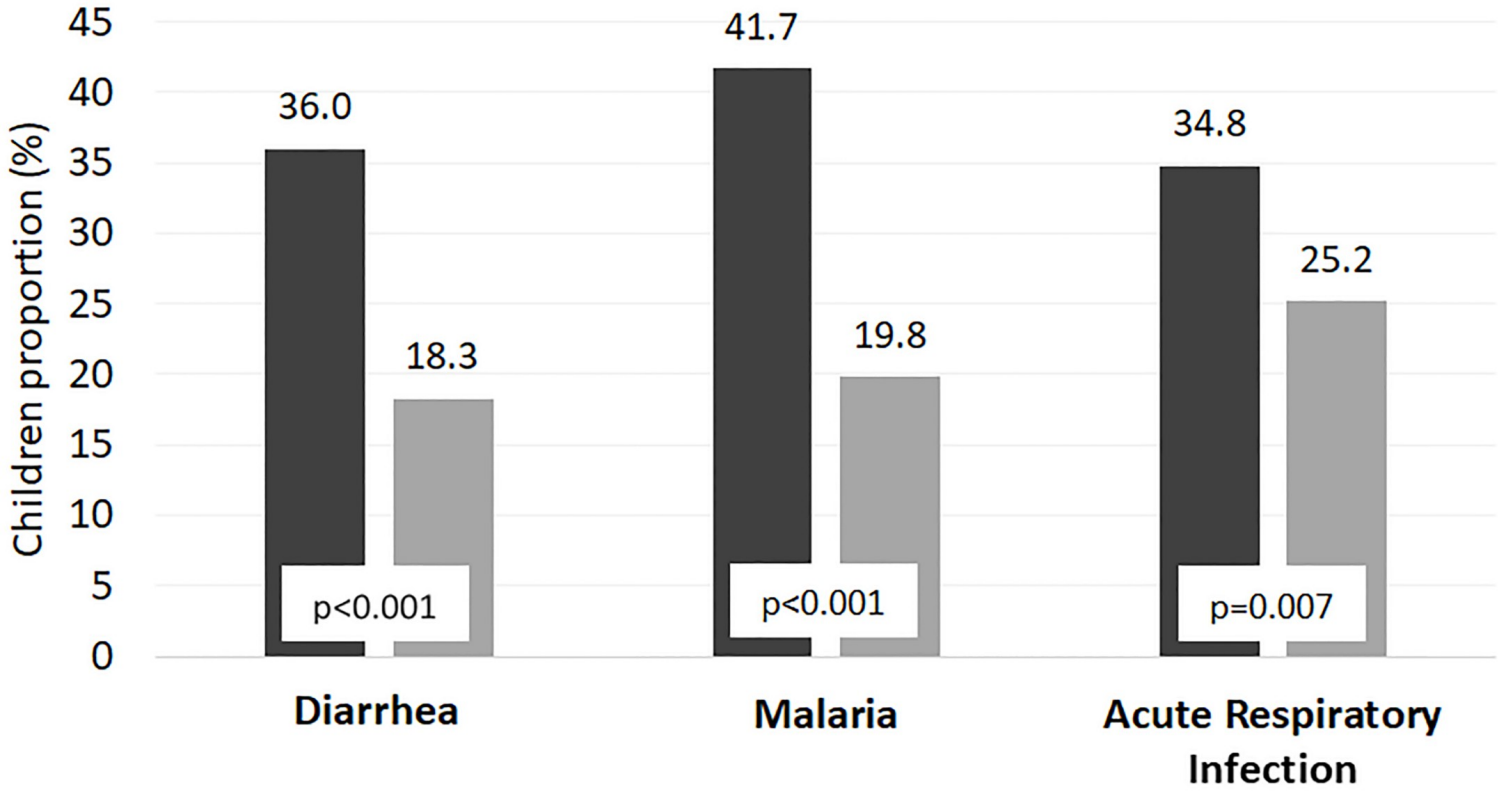
Proportion of other diseases detected during outpatient treatment of severe acute malnutrition compared between the two models.

In the children who recovered, the CHW group had a median length of stay of 39 days (IQR 29–51), and the HF group had a median length of stay of 42 days (IQR 28–49), with no significant difference (p = 0.740) between the two. However, slightly more children in the HF group (54% vs. 47%; p = 0.073) than in the CHW group remained in treatment for more than 40 days (almost 6 weeks), which is the median stay considering both groups together. The linear regression analyses were adjusted by age, included all 930 children admitted to treatment, and showed an inverse association between WHZ at admission and length of stay (B coefficient = -1.985, 95% C.I. [-3.517–-0.453], p = 0.011), although the MUAC at admission did not show this same association (p = 0.185). The proportion of children who were absent for one or more nonconsecutive visits during treatment was higher in the HF group (25.9% vs. 17.0%, p = 0.003; OR adjusted by sex, age and key variables that differed at admission as edema, relapses and readmissions = 1.534, 95% C.I. [1.059–2.222], p = 0.024) than in the CHW group. Fig 3 shows that the other signs and diseases assessed during the SAM treatment (diarrhea, malaria, and acute respiratory infection) were more prevalent in the CHW group than in the HF group.

The results at discharge are shown in Table 3. The proportion of cured children was lower in the HF group than in the CHW group (88.7% vs. 95.9%), and the probability of not being cured in the HF group at the end of the treatment was 3.3 times higher than that in the CHW group after adjusting for sex, age, and conditions at admission. The proportion of children who defaulted from treatment was also higher in the HF group (9.8% vs. 3.7%), and the children’s probability of being discharged because of default was more than three times higher in the HF group than in the CHW group. The probability of death did not differ significantly even though the proportion of fatal cases in the HF group was almost triple that in the CHW group (1.5% vs. 0.4%). Only three children in the CHW group were discharged as nonresponsive (0.6%). Nonresponder rates between the two groups could not be compared, as this data was not included in the national protocols, nor was it collected at the HF level. No significant difference was found in the proportion of children who had to be referred to nutritional stabilization centers during treatment due to complications (HFs 7.2% vs CHWs 9.5%, p = 0.224).

**Table 3:** Treatment outcomes considering the two models of severe acute malnutrition outpatient management.

	Community Health Workers		Health Facilities			
**Discharge Outcomes**^a^	n = 487		n = 336		OR [95% C.I.]^b^	p value
	n	%	n	%		
Cure	467	95.9	298	88.7	3.311 [1.772–6.185]	<0.001
Default	18	3.7	33	9.8	3.345 [1.702–6.577]	<0.001
Death	2	0.4	5	1.5	2.748 [0.579–13.043]	0.308^NS^
**Anthropometric improvement in cured children**^c^	n = 466		n = 295		n = 466	
	Median	IQR	Median	IQR		
Weight gain						
Total (kg)	1.50	1.20–1.80	1.50	1.20–1.80	0.577^NS^	
Per day (gr/kg)	5.45	3.63–7.52	5.45	3.63–7.52	0.976^NS^	
MUAC gain						
Total (mm)	13.00	10.00–13.00	13.00	10.00–13.00	0.050^NS^	
Per day (mm)	0.33	0.22–0.50	0.33	0.22–0.50	0.050^NS^	

IQR: Inter-Quartile Range; NS: Not significant; OR: Odds Ratio; SD: Standard deviation

^a^ Chi-square test comparing proportions among discharge outcomes between models: p<0.001

^b^ Cochran-Mantel-Haenszel test adjusted by sex, age and the key variables with differences at admission (edema, relapse and readmission) considering the Health Facility model over the Community Health Worker model (RR of not being cured, RR of defaulting and RR of death)

^c^ Calculated according to standardized indicators for CMAM programs [20]; excluding those children with edema at admission.

^d^ Nonparametric Mann-Whitney test was applied based on the nonnormal distribution of the variables.

Table 3 also summarizes the evolution in the anthropometric measurements of the children discharged as cured; no significant differences were found between the two models. Finally, when including all children regardless of their treatment provider (n = 930), adjusted regression analyses proved that increased WHZs and MUACs at admission (less severe condition) are associated with a higher probability of being cured after treatment (Table 4).

**Table 4:** Association of anthropometric measurements at admission with the probability of cure at discharge.

	Unadjusted univariate logistic regression analysis		Adjusted univariate logistic regression analysis*	
	β coefficient [95% C.I.]	p value	β coefficient [95% C.I.]	p value
MUAC	1.052 [1.027–1.078]	<0.001	1.074 [1.047–1.102]	<0.001
WHZ	1.519 [1.242–1.858]	<0.001	1.603 [1.292–1.988]	<0.001

C.I.: Confidence Interval; MUAC: Middle-Upper Arm Circumference; WHZ: Weight for Height Z-score.

*Adjusted by sex, age and key conditions at admission (edema, readmission and relapse).

## Discussion

According to recent assessments in a rural region of Mali, malnutrition is the main avoidable factor related to child mortality [21]. This study shows that children receiving SAM treatment from CHWs generally exhibited a less severe stage at admission, with lower rates of edema and better anthropometric condition compared to children treated at HFs. A previous analysis on the cost-effectiveness of this new approach for SAM management with CHWs as treatment providers showed that families can save half the time and one-third of the money that it would cost to treat their children at the HFs [22]. This cost-saving could prevent families from delaying the initiation of required nutritional treatment, allowing children to be admitted in a relatively less severe condition.

The results also show that increased MUACs and WHZs at admission were associated with an increased probability of successful recovery. This is consistent with other publications assessing these two models of treatment in Malawi [23]. The Malawi study also found that children with relatively low anthropometric measurements at admission were more likely to default from the program or achieve poor clinical outcomes. A recent systematic review found that the strongest and most consistent risk factor for relapse after SAM treatment in CMAM programs is low anthropometric measurements at admission [24]. Another study in India reported that children with a MUAC under 105 mm at admission had a 6.46 times increased risk of SAM a year after discharge, although they did not find any significant association with a low WHZ [25]. Various studies have found evidence that high severity at admission is associated with an increased risk of death after discharge. In Bangladesh, it was found that severe wasting, considered a WHZ <-4, was associated with a 3.64 times higher probability of death three months after the end of treatment [26]. In the same setting, Kerac et al. [27] showed that the death hazard ratio 90 days after discharge decreased with each increased anthropometric measurement unit at admission (HR adjusted for age, edema, and HIV status; 0.80 per cm of MUAC and 0.75 per WHZ unit). Evidence of this relationship between severity at admission and mortality after discharge has also been found for other anthropometric indicators of growth and nutritional status, such as weight-for-age, height-for-age, and head circumference [26–28].

Studies on the other diseases managed under the iCCM protocol have previously demonstrated this reduction in the risk of morbidity and mortality based on early treatment. A systematic review concluded that pneumonia managed by CHWs was associated with a 32% reduction in mortality [29], and other studies have reported that this figure increased to 70% [30]. Similar results have been reported for the early treatment of malaria in several countries [31]. This study found that CHWs detected and treated more cases of infectious diseases both at admission and during treatment than HFs, with malaria detection and treatment showing the largest difference between the two groups. The decreased rates in the HF group could be related to the medical staff’s high workload, which prevents the adequate allocation of time to assess other medical signs beyond acute malnutrition. Similar challenges have been reported with HIV testing in Malawi, where centers implementing CMAM were often overwhelmed by the workload involved in managing acute malnutrition [32]. On the other hand, a previous study on an iCCM+ program in Bangladesh demonstrated that although including SAM treatment in the package of interventions delivered by CHWs increased their overall workload by an estimated three hours per week, this increase did not negatively affect the quality of care in the other preventive and curative services delivered [33]. However, further time allocation studies comparing the performance of HFs and CHWs are required.

In relation to the treatment duration of the recovered children, this study did not find a significant difference between both models of service delivery. However, an increased WHZ at admission was associated with a reduced length of stay. A study conducted in Ethiopia with 420 children found that starting treatment with a MUAC above 106 mm was significantly associated with a reduction in the amount of time needed to recover, but the decrease was only from 27 to 24 days [34]. In contrast, another study carried out in Burkina Faso involving 22,094 children showed that MUAC at admission was inversely associated with length of stay; specifically, it found that children admitted with a MUAC <100 mm stayed for 46.6 days, while those with a MUAC between 116–118 stayed an additional ten days on average [35]. The study in Burkina Faso found an overall treatment duration of 52 days for children admitted with a MUAC <115 mm or WHZ <-3, which is higher than the 42 days registered by the HFs in this study. The same length of stay (42 days) has been reported in other studies in Ethiopia, but they were based on a discharge criterion of 15% weight gain [36, 37]. One of them found that considering MUAC, a discharge criterion could mean increased length of stays, since they found that 20.6% of the children who showed a 15% weight gain would still be considered acutely malnourished if a MUAC <115 mm criterion was applied [37].

The aforementioned iCCM+ study in Bangladesh [38] reported a length of stay of 37.4 ± 0.6 days, which is close to the 39 found in this study. However, the discharge criteria (MUAC >110 mm and 15% weight gain) were different than those required by the Malian Health Ministry policy (MUAC >125 mm or WHZ > -1.5). Nevertheless, the anthropometric improvement during treatment in both groups in this study was similar to what was reported in the Bangladesh study (weight: 6.7±0.1 g/kg/day; MUAC: 0.4 mm/day). A systematic review of the recovery of edematous children from inpatient and outpatient programs registered a weight gain after loss of edema of 2.7 to 4.0 g/kg/day and a MUAC gain of 0.2 to 0.3 mm/day [39], both of which were lower than those in this study. That review also concluded that the worst outcomes were observed in studies where the children received inpatient care for the first week, supporting the idea that severity at admission has an influence on recovery. Previous studies on therapeutic feeding programs have also demonstrated that the most severe kwashiorkor patients gain less weight on average than marasmic patients (2.70 g/kg/day and 3.16 g/kg/day, respectively) [36].

In relation to discharge outcomes, both models achieved the Minimum Standards in Humanitarian Response (<75% recovery, <10% death, and <15% default) [40]. However, compared to the CHW model of treatment, children treated at HFs had a higher probability of missing one or more visits during treatment, with a three times higher risk of ultimately defaulting from the program and a total risk of not being cured that was also 3.3 times higher. Proximity to treatment has previously been identified as a factor influencing recovery in CMAM programs [41, 42]. The results of this study show that increased anthropometric measurements at admission are directly associated with an increased probability of being cured after treatment. The combination of early treatment and close follow-up due to the proximity of CHWs performing integrated management of childhood diseases could be the reason for the high effectiveness of iCCM+ interventions compared to the treatment provided at health centers [9]. Furthermore, the recovery rate achieved by CHWs in this study (95.5%) was the highest of all the iCCM+ experiences published to date, which range from 60.0% in India to 93.8% in Angola [8,43]. Only one study in Ethiopia found that treatment by CHWs was associated with worse outcomes than treatment at HFs, with a 1.5 times higher probability of nonrecovery [44].

There were limitations to this study. As mentioned above, this was a secondary analysis of data recorded for a study with a different design in which HFs plus CHWs were part of the intervention area, and only some of the HFs were part of the control area. Moreover, follow-up weight and height were not recorded, limiting the analysis of anthropometric evolution during treatment. Further studies assessing the long-term status of children who recovered with each treatment delivery model are needed to ascertain whether the iCCM+ approach has an effect on reducing relapse and mortality rates over time.

## Conclusions

The addition of SAM treatment into the curative tasks provided closer to the families by the CHWs can result in a reduction in the severity at admission and fewer absences and defaults during the treatment compared to standard CMAM care provided at health facilities generally located farther away. CHWs also detected and treated more cases of diarrhea, malaria, and acute respiratory infection, which allows SAM to be tackled in a more integrated manner. Accordingly, CHWs achieve better discharge outcomes with higher recovery rates and fewer defaulters than treatment provided by medical staff at health centers.

## Supporting information

S1 Data (XLSX)

## Abbreviations

CHWs: Community Health Workers
CMAM: Community Management of Acute Malnutrition
HFs: Health Facilities
iCCM: integrated Community Case Management
INRSP: *Institute National de la Recherche en Santé Publique*
MUAC: Mid-Upper Arm Circumference
RUTF: Ready-to-Use Therapeutic Food
SAM: Severe Acute Malnutrition
WHZ: Weight-for-Height z-score

## References

[1] United Nations Children’s Fund (UNICEF). Severe Acute Malnutrition [internet]. 2015. [cited 30 may 2018]. https://www.unicef.org/nutrition/index_sam.html

[2] Olofin I, McDonald CM, Ezzati M, Flaxman S, Black RE, Fawzi WW, et al Associations of suboptimal growth with all-cause and cause-specific mortality in children under five years: a pooled analysis of ten prospective studies. PLoS ONE. 2013; 8(5): e64636 10.1371/journal.pone.0064636 23734210 PMC3667136

[3] United Nations Children’s Fund (UNICEF), World health Organization (WHO), the World Bank Group (WGB). Joint child malnutrition estimates. Levels and trends in child malnutrition. Key findings of the 2019 edition. [internet]. 2019. [cited 20 may 2019]. https://www.who.int/nutgrowthdb/estimates2018/en/

[4] Collins S, Dent N, Binns P, Bahwere P, Sadler K, Hallam A. Management of severe acute malnutrition in children. The Lancet. 2006; 368 (9551): 1992–2000. 10.1016/S0140-6736(06)69443-9 17141707

[5] United States Agency for International Development (USAID). Training guide for community-based management of acute malnutrition (CMAM). Module 4. Outpatient care for the management of Severe Acute Malnutrition without complications. Food and Nutrition Technical Assistance (FANTA III) [internet]. 2018. [cited 30 may 2019]. https://www.fantaproject.org/focus-areas/nutrition-emergencies-mam/cmam-training

[6] Rogers E, Myatt M, Woodhead S, Guerrero S, Álvarez JL. Coverage of community-based management of severe acute malnutrition programmes in twenty-one countries, 2012–2013. PLoS ONE. 2015; 10 (5): e0128666. 26042827 10.1371/journal.pone.0128666 PMC4456359

[7] Lenters LM, Wazny K, Webb P, Ahmed T, Buhtta ZA. Treatment of severe and moderate acute malnutrition in low- and middle-income settings: a systematic review, meta-analysis and Delphi process. BMC Public Health. 2013; 13 (Suppl 3): S23. 24564235 10.1186/1471-2458-13-S3-S23 PMC3847503

[8] Young M, Wolfheim C, Marsh DR, Hammamy D. World Health Organization/United Nations Children’s Fund Joint Statement on Integrated Community Case Management: an equity-focused strategy to improve access to essential treatment services for children. Am J Trop Hyg, 2012; 87 (Suppl.5): 6–10. 10.4269/ajtmh.2012.12-0221 PMC3748523 23136272

[9] López-Ejeda N, Charle-Cuellar P, Vargas A, Guerrero S. Can Community Health Workers manage uncomplicated severe acute malnutrition? A review of operational experiences in delivering SAM treatment through community health platforms. Mat Child Nutr. 2019; 15(2): e12719. 10.1111/mcn.12719 PMC6587873 30315743

[10] Puett C, Sadler K, Alderman H, Coates J, Fledler JL, Myatt M. Cost-effectiveness of the community-based management of severe acute malnutrition by community health workers in southern Bangladesh. Health Policy Plann, 2013; 28, 386–399. 10.1093/heapol/czs070 22879522

[11] Rogers E, Martínez M, Álvarez-Morán JL, Alé FGB, Charle P, Guerrero S, et al Cost-effectiveness of the treatment of uncomplicated severe acute malnutrition by community health workers compared to treatment provided at an outpatient facility in rural Mali. Hum Resour Health, 2018; 16, 12 10.1186/s12960-018-0273-0 29458382 PMC5819265

[12] United Nations Office for the Coordination of Humanitarian Affairs (OCHA). Mali Humanitarian Response Plan. January-December 2018 [internet]. 2018. [cited 5 june 2019]. https://reliefweb.int/sites/reliefweb.int/files/resources/hrp-2018_mali_en_20180222_vf_0.pdf

[13] United Nations Development Programme (UNDP). Human development indices and indicators. 2018 statistical update [internet]. 2018. [cited 5 june 2019]. http://hdr.undp.org/sites/default/files/2018_human_development_statistical_update.pdf

[14] Institut National de la Statistique République du Mali (INSTAT). Enquête Nationale Nutritionnelle Anthropométrique et de mortalité rétrospective suivant la méthodologie SMART, Mali 2017.[internet]. 2018. [cited 5 june 2019]. http://instat-mali.org/contenu/eq/rafsmart17_eq.pdf

[15] Ministère de la Santé et de L’Hygiène Publique et Direction National de la Santé, République du Mali. Soins Essentiels dans la Communauté. Guide national pour la mise en œuvre; 2015.

[16] Álvarez-Morán JL, Alé FGB, Charle P, Sessions N, Doumbia D, Guerrero S. The effectiveness of treatment for severe acute malnutrition (SAM) delivered by community health workers compare to a traditional facility based model. BMC Health Serv Res. 2018; 18: 207 10.1186/s12913-018-2987-z 29580238 PMC5870488

[17] Álvarez-Morán JL, Alé FGB, Rogers E, Guerrero S. Quality of care for treatment of uncomplicated severe acute malnutrition delivered by community health workers in a rural area of Mali. Mat Child Nutr. 2018; 14: e12499. 10.1111/mcn.12449 PMC6866144 28378463

[18] World Health Organization (WHO). Updates on the management of Severe Acute Malnutrition in Infants and Children. Guideline. [internet]. 2013. [cited 30 may 2019]. https://apps.who.int/iris/bitstream/handle/10665/95584/9789241506328_eng.pdf?sequence=1 24649519

[19] World Health Organization (WHO). Multicentre Growth Reference Study Group. WHO Child Growth Standards: Length/height-for-age, weight-for-age, weight-for-length, weight-for-height and body mass index-for-age: Methods and development. Geneva, World Health Organization. [internet]. 2007. [cited 30 may 2019]. http://www.who.int/childgrowth/standards/technical_report/en/index.html

[20] Navarro-Colorado C, Andert C, Mates E, Vazquez L, Martín J, Shoham J, et al. Standard Indicators and categories for better CMAM reporting. April 2015 edition. Save the Children and Humanitarian Innovation Fund. [internet]. 2015. [cited 5 june 2019]. http://www.cmamreport.com/cmam/downloads#sig

[21] Wilcox ML, Kumbakumba E, Diallo D, Mubangizi V, Kirabira P, Nakaggwa F, et al Circumstances of child deaths in Mali and Uganda: a community-based confidential enquiry. Lancet Glob Health. 2018; 6: e691–702. 10.1016/S2214-109X(18)30215-8 29773123

[22] Rogers E, Martínez K, Álvarez-Morán JL, Alé F.G.B., Charle P, Guerrero S, et al Cost-effectiveness of the treatment of uncomplicated severe acute malnutrition by community health workers compared to treatment provided at an outpatient facility in rural Mali. Hum Resour Health. 2018; 16: 12 10.1186/s12960-018-0273-0 29458382 PMC5819265

[23] Linneman Z, Matlisky D, Ndekha MD, Manary MJ, Maleta K, Manary MJ. A large-scale operational study of home-based therapy with ready-to-use therapeutic food in childhood malnutrition in Malawi. Mat Child Nut. 2007; 3 (3): 206–15. 10.1111/j.1740-8709.2007.00095.x PMC6860523 17539889

[24] Stobaugh HC, Mayberry A, McGrath M, Bahwere P, Zagre NM, Manary MJ, et al Relapse after severe acute malnutrition: A systematic literature review and secondary data analysis. Mat Child Nutr. 2019; 15 (2): e12702. 10.1111/mcn.12702 PMC6587999 30246929

[25] Pandey P, Jain S, Sharma A. How healthy are children one year after discharge from nutritional rehabilitation centres?. Trop Doct. 2018; 48 (4): 277–82. 10.1177/0049475518786854 30012079

[26] Chisti MJ, Graham SM, Duke T, Ahmed T, Faruque ASG, Ashraf H, et al Post-discharge mortality in children with severe malnutrition and pneumonia in Bangladesh. PLoS ONE. 2014; 9(9): e107663 10.1371/journal.pone.0107663 25225798 PMC4167196

[27] Kerac M, Bunn J, Chagaluka G, Bahwere P, Tomkins A, Collins S, et al Follow-up of post-discharge growth and mortality after treatment for severe acute malnutrition (FuSAM study): a prospective cohort study. PLoS ONE. 2014; 9(6): e96030 10.1371/journal.pone.0096030 24892281 PMC4043484

[28] John C, Diala U, Adah R, Lar L, Envuladu EA, Adedeji I, et al Survival and nutritional status of children with severe acute malnutrition, six months post-discharge from outpatient treatment in Jigawa state, Nigeria. PLoS ONE. 2018; 13(6): e0196971 10.1371/journal.pone.0196971 29924797 PMC6010258

[29] Das JK, Lassi ZS, Salam RA, Bhutta ZA. Effect of community based interventions on childhood diarrhea and pneumonia: uptake of treatment modalities and impact on mortality. BMC Pub Health. 2013; 13 (suppl. 3): S29. 24564451 10.1186/1471-2458-13-S3-S29 PMC3953053

[30] Theodoratou E, Al-Jilaihawi S, Woodeard F, Ferguson J, Jhass A, Balliet M, et al The effect of case management on childhood pneumonia mortality in developing countries. Int J Epidemiol. 2010; 39 (Suppl. 1): i155–71. 20348118 10.1093/ije/dyq032 PMC2845871

[31] Smith-Paintain L, Willey B, Kedenge S, Sharkey A, Kim J, Buj V, et al Community Health Workers and stand-alone or integrated case management of malaria: a systematic literature review. Am J Trop Med Hyg. 2014; 91 (3): 461–70. 10.4269/ajtmh.14-0094 24957538 PMC4155545

[32] Chitete L, Puoane T. What Health Service Provider Factors Are Associated with Low Delivery of HIV Testing to Children with Acute Malnutrition in Dowa District of Malawi?. PLoS ONE. 2015; 10 (5): e0123021 10.1371/journal.pone.0123021 25933164 PMC4416721

[33] Puett C, Coates J, Alderman H, Sadler K. Does greater workload lead to reduced quality of preventive and curative care among community health workers in Bangladesh? Food Nut Bull. 2012; 33(4): 273–85. 10.1177/156482651203300408 23424894

[34] Gebremichael DY. Predictors of nutritional recovery time and survival status among children with severe acute malnutrition who have been managed in therapeutic feeding centers, Southern Ethiopia: retrospective cohort study. BMC Pub Health. 2015; 15:1267. 26689192 10.1186/s12889-015-2593-5 PMC4687080

[35] Goossens S, Bekele Y, Yun O, Harczi G, Ouannes M, Shepherd S. Mid-Upper Arm Circumference Based nutrition programming: evidence for a new approach in regions with high burden of acute malnutrition. PLoS ONE. 2012; 7 (11): e49320 10.1371/journal.pone.0049320 23189140 PMC3506602

[36] Collins S, Sadler K. Outpatient care for severely malnourished children in emergency relief programmes: a retrospective cohort study. Lancet. 2002; 360: 1824–30. 10.1016/S0140-6736(02)11770-3 12480359

[37] Mengesha MM, Deyessa N, Tegegne BS, Dessie Y. Treatment outcome and factors affecting time to recovery in children with severe acute malnutrition treated at outpatient therapeutic care program. Glob Health Act. 2016; 9 (1): 30704. 10.3402/gha.v9.30704 PMC4939403 27396484

[38] Sadler K, Puett C, Mothabbir C, Myatt M. Community case management of severe acute malnutrition in Southern Bangladesh. Save the Children, Feinstein International Center, Tufts, University. [internet] 2011. [cited 25 june 2019]. http://fic.tufts.edu/publication-item/community-case-management-of-severe-acute-malnutrition-in-southern-bangladesh/

[39] Roberfroid D, Hammami N, Mehta P, Lachat C, Verstraeten R, Prinzo ZW, et al. Management of oedematous malnutrition in infants and children aged > 6 months: a systematic review of the evidence. World Health Organization [internet]. 2013. [cited 25 june 2019]. http://www.who.int/nutrition/publications/guidelines/updates_management_SAM_infantandchildren_review2.pdf

[40] The Sphere Project. Minimum standards in food security and nutrition. In: Humanitarian charter and minimum standards in humanitarian response [internet]. 2018. [cited 25 june 2019]. https://www.spherestandards.org/

[41] Massa D, Woldemichael K, Tsehayneh B, Tesfay A. Treatment outcome of severe acute malnutrition and determinants of survival in Northern Ethiopia: A prospective cohort study. Int J Nutr Metab. 2016; 8 (3): 12–23.

[42] Kabalo MY, Seifu CN. Treatment outcomes of severe acute malnutrition in children treated within Outpatient Therapeutic Program (OTP) at Wolaita Zone, Southern Ethiopia: retrospective cross-sectional study. J Health Popul Nut. 2017; 36: 7. 10.1186/s41043-017-0083-3 PMC5345228 28279227

[43] Goudet S, Jayaraman A, Chanani S, Osrin D, Devleesschauwer B, Bogin B, et al Cost effectiveness of a community based prevention and treatment of acute malnutrition programme in Mumbai slums, India. PLoS ONE. 2018; 13(11): e0205688 10.1371/journal.pone.0205688 30412636 PMC6226164

[44] Shanka NA, Lemma S, Abyu DM. Recovery rate and determinants in treatment of children with severe acute malnutrition using outpatient therapeutic feeding program in Kamba District, South West Ethiopia. J Nutr Disorders Ther. 2015; 5:2.

